# Harvest effects on density and biomass of *Neopicrorhiza scrophulariiflora* vary along environmental gradients in the Nepalese Himalayas

**DOI:** 10.1002/ece3.5355

**Published:** 2019-06-19

**Authors:** Mukti Ram Poudeyal, Henrik Meilby, Bharat Babu Shrestha, Suresh Kumar Ghimire

**Affiliations:** ^1^ Central Department of Botany Tribhuvan University Kathmandu Nepal; ^2^ Department of Food and Resource Economics University of Copenhagen Frederiksberg C Denmark

**Keywords:** clonal herb, conservation, elevation gradient, medicinal plant, shrub facilitation, trade

## Abstract

A surprisingly large number of species potentially threatened by human harvest lack quantitative ecological studies incorporating harvest effects, especially clonal species in the alpine Himalayas. We studied density and biomass variation of a threatened medicinal herb, *Neopicrorhiza scrophulariiflora,* to examine the effect of harvest on plant performance. The study covered two regions with contrasting harvest situations—one with open‐access and another protected from commercial harvesting. Four populations from each region were compared along an elevation gradient (3,800–4,800 m). Also, we conducted in situ interviews with 165 and 38 medicinal and aromatic plant users in open‐access and protected regions, respectively, to assess the collection and use patterns of the target species. The quantity harvested per household for traditional healthcare use was similar in both regions. We found no evidence of trade‐driven collection in the protected region but in the open‐access region a trade‐based annual collection of 35–465 kg dried rhizomes per household had a strong negative effect on both density and biomass. In the protected region, the effect of harvest intensity on plant density was positive for vegetative and negative for reproductive individuals, whereas in the open‐access region, the effect was negative for both vegetative and reproductive individuals. The results indicated that a low harvest intensity had no adverse impact on *N. scrophulariiflora* populations; however, quantification of the optimum level of harvest remains to be explored. Shrub vegetation appeared to buffer the harvest impact on plant density, possibly through the retention of additional moisture. To maintain population viability, we suggest regulating harvest, for example, by introducing rotational harvest systems, ensuring that a sufficient number of reproductive individuals are left as a source of propagules in each harvested population and that populations are given time to recover between harvests.

## 1 INTRODUCTION

Plant life in high mountains, such as the Himalayas, is constrained by biotic and abiotic stress. Contemporary ecological studies from alpine regions primarily emphasize extreme abiotic conditions where plant growth is constrained by short growing season, low temperature, and limited resources (Dvorský et al., 2015; Klimešová, Jiří, Prach, & Košnar, 2012). Interactions with biotic factors, including herbivory, habitat competition, and facilitation, and anthropogenic activities are issues that are not well addressed in plant population studies from alpine areas in the Himalayas (but see Chu et al., 2008, 2009; Ghimire, Gimenez, Pradel, McKey, & Aumeeruddy‐Thomas, 2008) and elsewhere (Callaghan, Carlsson, Jónsdóttir, Svensson, & Jonasson, 1992; Körner, 2003). Alpine plant species in the Himalayas are mostly perennial and many of them exhibit clonal growth (Klimeš, 2003; Klimešová, Doležal, Dvorský, Bello, & Klimeš, 2011). Clonal units in such species not only share resources and photosynthates but also absorb negative effects of physical stress, competition, and herbivory, enabling alpine plants to respond to environmental changes (Klimešová et al., 2011; Lei, 2010). At habitat level, the inter‐species competition is overlaid by the facilitative role of surrounding vegetation (Ballantyne & Pickering, 2015; Wang, Liang, & Wang, 2014). Delivery of shade and shelter, moisture retention, nutrient accumulation, temperature regulation, and defense against herbivory and physical damage are important services that can be offered by the surrounding vegetation (Ballantyne & Pickering, 2015; Callaway et al., 2002). Thus, the balance between facilitative and competitive environmental interactions has significant ecological implications to the performance of alpine plant species (Chu et al., 2009; Klimešová et al., 2012).

In addition to environmental interactions, alpine plants are in many cases also subjected to high levels of anthropogenic impacts. In the Himalayas, for example, international trade in medicinal and aromatic plants (MAPs) (Olsen, 2005; Pyakurel, Sharma, & Smith‐Hall, 2018) has created extreme pressure on populations of some alpine plants and their habitats. In addition, other factors, such as livestock grazing and fire, may reduce densities of alpine plant populations (Niu, He, Zhang, & Lechowicz, 2016). Harvesting of whole plants or plant parts may affect reproduction, survival, and growth, thereby affecting plant population dynamics (Gaoue, Horvitz, Ticktin, Steiner, & Tuljapurkar, 2013; Ghimire et al., 2008; Ghimire, McKey, & Aumeeruddy‐Thomas, 2005; Huai, Wen, Xu, & Bai, 2013; Ticktin, 2004). The extent of harvest impact on plant populations, however, varies depending on habitat conditions, plant growth strategies, and species‐specific processes, such as regeneration and colonization (Gaoue et al., 2013; Ticktin, 2004, 2015). Clonal plant species to some extent buffer the anthropogenic effects like harvesting by mobilizing stored reserves to maintain normal phases of growth (Mandle & Ticktin, 2012). Furthermore, the clonal tendency toward developing a bud bank after a disturbance also plays a crucial role in vegetative reproduction (Evette, Bédécarrats, & Bornette, 2009). The clonal architecture, especially in plants exhibiting a “guerilla” strategy of clonal growth, producing long and spreading vegetative offshoots, allows the plant to escape stressful microsites and colonize more suitable ones (Ghimire et al., 2005; Humphrey & Pyke, 1998). In clonal herbs exhibiting a “guerilla” strategy, the density of plants is positively affected by light harvesting if it occurs at sufficiently long intervals and does not exceed the regeneration potential (Ghimire et al., 2005). Thus, with responsible management practices, vegetative propagation in clonal plant species could compensate for reduced biomass caused by anthropogenic disturbances.

The interactions between people and plants in the Himalayas are not yet fully understood (but see Ghimire et al., 2005; Ghimire et al., 2008; Rokaya, Münzbergová, & Dostálek, 2017). Specifically, determining the relative effects of habitat factors, such as substrate, vegetation cover, and disturbance on plant density and biomass is crucial for development of well‐informed and effective conservation policies. This study aims to disentangle the factors that control the density and biomass of a clonal medicinal herb, *Neopicrorhiza scrophulariiflora* (Pennell) D.Y. Hong, across a continuum of environmental conditions and harvest intensities in the alpine Himalayas, Nepal. This species is highly threatened due to overharvesting of its rhizome for regional and international trade (Ghimire et al., 2005). Studies emphasizing the variation in density and biomass of such species across a continuum of environmental conditions along an elevation gradient could enhance our understanding of the ability of the species to respond to environmental stress. We will address three questions: (a) In what ways do plant utilization patterns vary between study regions in terms of the purpose and intensity of harvest, and whether harvest takes place in the beginning or at the end of the growing season? (b) How do elevation, surface and vegetation cover, and anthropogenic disturbance influence the density and biomass of *N. scrophulariiflora*? and (c) Does plant response to harvest impact vary between study regions? To answer these questions, we first assessed plant utilization systems and analyzed density, population structure, and biomass variation in different populations along elevation gradients in two study areas characterized by different protection regimes. The species' interaction with environmental factors, including habitat conditions, surrounding vegetation, and human harvest regimes, was analyzed to evaluate these associations.

## 2 MATERIALS AND METHODS

### 2.1 Study area

**Figure 1:**
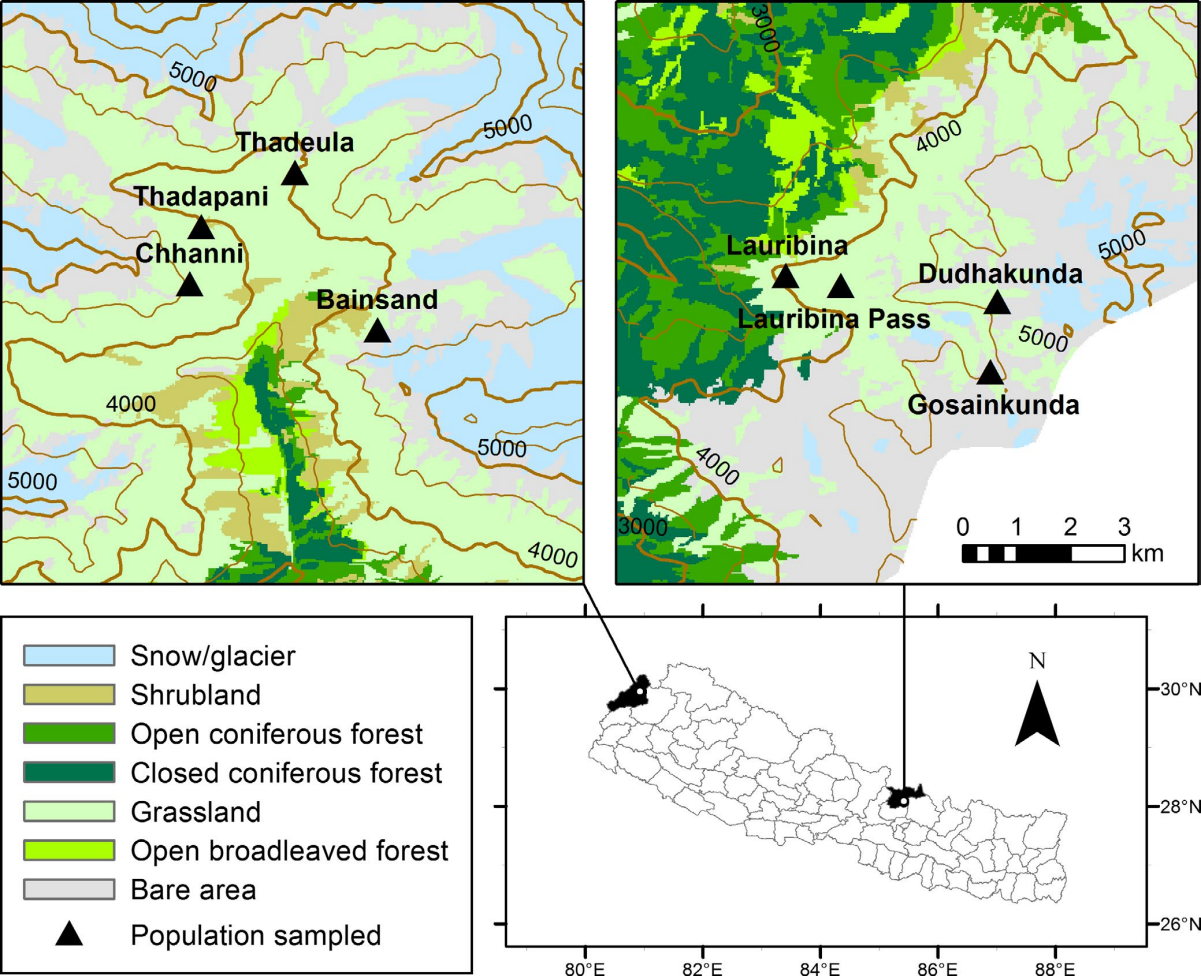
Map of the study area: triangles indicate populations included for the ecological study in Api‐Nampa Conservation Area (ANCA, left) and Langtang National Park (LNP, right). The thin and thick contour lines are provided to define the elevation scale in every interval of 500 m and 1,000 m from 3,000 to 5,000 m (source of land cover map: Uddin et al., 2015)

The study was conducted in two protected areas (hereafter referred to as regions): Api‐Nampa Conservation Area (ANCA) and Langtang National Park (LNP) in the north‐western and north‐central parts of Nepalese Himalayas (Figure 1). Both protected areas in those regions are managed by the Department of National Parks and Wildlife Conservation of the Government of Nepal. The Community‐led Management Council for ANCA (located in Darchula District) and the national park office for LNP (in Rasuwa District) are the management bodies responsible at the local level. The climate in both regions varies from subtropical to alpine‐nival. The two regions are similar in terms of annual precipitation (average of 2,100 mm in ANCA, and 2,300 mm in LNP) and annual average temperature (range: 4–27°C for ANCA and 2–25°C for LNP; both precipitation and temperature were recorded at the nearest meteorological stations located at 1,900 and 1,100 m above sea level, respectively). Both regions are characterized by high mountains and rugged terrain. Almost 70% of residents in ANCA and about 50% in LNP live below the poverty line (Pyakurel et al., 2018; Uprety, Poudel, Asselin, Boon, & Shrestha, 2011). Local livelihoods depend on traditional agriculture, livestock rearing, and seasonal labor (CBS, 2012). The upper slopes are fragile and arable fields are limited to low elevations (<3,000 m), but the productivity is lower in ANCA than in LNP (CBS, 2012; DDC, 2015; Fox, Podger, & Yonzon, 1996). Therefore, people in ANCA supplement their income mostly by harvesting and selling high‐valued MAPs and other forest products (Pouliot, Pyakurel, & Smith‐Hall, 2018; Pyakurel et al., 2018). In LNP, people primarily supplement their income through livestock rearing, cheese production, and tourism, and are less involved in harvesting and trade of forest products (Shakya, 2016).

Api‐Nampa Conservation Area was recently established in 2010, and in this region commercial harvesting of MAP is legally permitted. The ANCA Management Council issues collection and transportation permits for MAPs (GoN, 2015). Langtang National Park has been protected from commercial harvesting of forest products since its establishment in 1976, and to date, trade permissions have not been issued for any products, except for a high‐value medicinal caterpillar fungus, *Ophiocordyceps* (*Ophiocordyceps sinensis* (Berk.) G. H. Sung et al.) (HMG/N, 1978; personal communication with the conservation officer, 26 July 2018). Owing to the differences in MAP harvest and trade regulations, we hereafter refer to ANCA as “open” and LNP as “protected.”

### 2.2 Study species

*Neopicrorhiza scrophulariiflora* (Pennell) D.Y. Hong (hereinafter referred to as *Neopicrorhiza*) of the family Plantaginaceae (previously Scrophulariaceae) (APG IV, 2016) is a perennial, rhizomatous herb that exhibits clonal growth. It is confined to the pan‐Himalayas, the Tibetan Plateau, Assam‐Burma, and south‐central China, and is found at elevations between 3,500 and 4,800 m (Ghimire et al., 2005; Hong, 1998). The plant is relatively slow growing and is mostly confined to cool north‐facing slopes (Shrestha & Jha, 2009). Flowering and fruiting occur in June–September. Seedling recruitment is low, and propagation mainly takes place by vegetative means (Ghimire et al., 2005). The modular growth of the individual genet takes place by horizontal extension of ramets (vegetative offshoots), which can survive independently when detached. The clonal growth of *Neopicrorhiza* has been described as a “guerrilla” strategy, leading to spaced clusters of spreading fugitive ramets connected below ground by horizontal rhizomes (Ghimire et al., 2005).

The rhizomes of *Neopicrorhiza* are valued for the treatment of cough, cold, headache, fever, high blood pressure, conjunctivitis, bile reflux, and intestinal pains, among others (Ghimire et al., 2005; Manandhar, 2002). It is one of the most commonly consumed herbs in the Himalayas and is appraised to have a high pharmacological potential (Li, Liu, Abdulla, Aisa, & Suo, 2014). The plant is resistant to grazing, and thus, premature and excessive harvesting is the main issue for its conservation (Ghimire et al., 2005; Shrestha & Jha, 2009). It is closely related to *Picrorhiza kurrooa* Royle, a species native to north‐west India listed in CITES Appendix II. Rhizomes of both species are traded globally, but *Neopicrorhiza* is more commonly traded than *P. kurrooa* (Mulliken, 2000). Rhizomes of *Neopicrorhiza* collected from Nepal are mostly traded to India in air‐dried form through well‐established marketing chains (Olsen, 2005) with a current annual trade volume of ca. 350 tons (personal communication with traders in ANCA and at a major road‐head collection center in Nepalgunj, south‐west Nepal, November/December 2017).

### 2.3 Interviews with local resource users

This work was carried out as a partial basis for the first author's Ph.D. dissertation at the Central Department of Botany (CDB), Tribhuvan University, Nepal. The study proposal was approved by the CDB Research Committee. Written permission for fieldwork was obtained from Department of National Park and Wildlife Conservation, Government of Nepal. The research objectives were explained, and prior informed consent was received from respondents before conducting interviews and consultations with MAP users in ANCA and LNP. The participants were informed they could withdraw their consent at any point in case of disagreement.

Commercial MAP harvesting and livestock grazing are the main human activities in the alpine regions of the Himalayas. Local people usually follow traditional systems of rotational grazing when livestock are brought to subalpine and alpine pastures for summer grazing. We recorded two types of MAP collectors: dedicated collectors, who collect only one target species at a time and are not involved in pastoralism; and opportunistic collectors, who collect different MAP species and are also involved in pastoralism (Olsen, 1998). On average across the grazing seasons of 2015–2017, we recorded 1,500 livestock (100 cattle and 1,400 sheep/goats) and 30 herders in ANCA, and 1,000 livestock (200 cattle and 800 sheep/goats) and 20 herders in LNP at the study locations (based on interviews with herders, 2015–2017). Commercial harvesting of *Neopicrorhiza* usually takes place during the late part of the growing season (August‐October). Apart from this, in ANCA, harvesting of *Neopicrorhiza* usually coincides with the harvesting season of *Ophiocordyceps* in late May to early July. We used in situ open interviews with 165 MAP users in ANCA and 38 in LNP to solicit information about harvesting and utilization. We assessed purpose and level of harvest of *Neopicrorhiza* by asking four questions: (a) Do you harvest *Neopicrorhiza*? (c) If yes, then for what purpose (only for domestic consumption or also for selling)? (c) Which season do you prefer to go for collection? and (d) How much quantity do you collect typically in a year?

### 2.4 Plot establishment and sampling

We carried out a sample‐based study in *Neopicrorhiza* populations during the peak growing season (June–August of 2015–2017). We identified four large populations of *Neopicrorhiza* in each of the two regions, within an elevation range of 3,500–4,800 m along upper Chameliya valley of ANCA and upper Trishuli watershed of LNP (Figure 1, Table S1). In each population, we laid out three transects at intervals of 100 m (Ghimire et al., 2008, 2005). In each transect, we established two plots (3 × 3 m) at horizontal distances of at least 20 m from each other, depending on field conditions. The clonal behavior of the study species is such that sprouting occurs readily from the parental ramet within a limited distance leading to a patchy form of distribution. Therefore, we established the plots subjectively where the plants occurred in sufficient number for sampling (Whittaker, 1956). We divided each plot into nine subplots (1 × 1 m). Among these, five subplots (four at the corners and one at the center) were systematically sampled and measured. In essence, the study included 48 plots with 240 subplots, and each of the samples in the two regions included half of these.

We studied plant density for ramet stage classes. In each subplot, we classified individual ramets, based on number and size of leaves and occurrence of reproductive organs, into one of the three stage classes (Oostermeijer, Brugman, Boer, & Nijs, 1996; Weppler, Stoll, & Stöcklin, 2006): (a) juvenile ramets bearing 1–4 smaller‐sized (range: 0.5–2.0 cm length) leaves; (b) adult vegetative ramets bearing more than four larger‐sized (>1.5–9.0 cm length) leaves but with no indication of sexual reproduction; and (c) reproductive ramets bearing any number and size of leaves and a reproductive peduncle. To describe population structure, we calculated the proportion of ramets in each stage class.

For the estimation of harvestable rhizome biomass, we asked local harvesters to collect 2–6 full‐grown ramets (adult vegetative and reproductive stages only) from eight to eighteen 1 × 1 m plots in each population, laid out randomly in the vicinity of plots used in density estimation. This way we collected biomass samples from 50 subplots in ANCA and 46 in LNP. In total, we extracted 161 biomass samples in ANCA and 133 in LNP (30–59 samples including above‐ and below‐ground parts per population). We measured the length and girth of each rhizome when fresh. Girth was calculated as a mean of three measurements at different nodal points along the rhizome. The volume of each rhizome was calculated based on girth and length assuming cylindrical shape. The biomass of each rhizome sample was measured after drying for 72 hr at 60°C in a hot air oven. The volume and biomass were used for calculation of rhizome tissue density (expressed in g/cm^3^). The total number of rhizomes per kilogram dry weight was calculated for each population based on the estimated mean biomass.

### 2.5 Plot‐based environmental variables

Geographical location (latitude and longitude) and topographic features (elevation, slope, and aspect) were measured for each plot. Latitude, slope, and aspect were used to calculate potential annual direct incident radiation (PADIR) (McCune, 2007; McCune & Keon, 2002). Vegetation data were recorded in each subplot (Table S2). The shrub canopy cover was estimated visually as total canopy cover of all shrub species, and shrub height was measured at three points located diagonally across the subplot. Cover of ground vegetation was estimated separately for herbs (all dicotyledons species, orchids, and lilies), graminoids (including all grasses and sedges), and lichens/mosses. Similarly, bare ground cover and rock cover were estimated for each subplot. Soil pH was measured using a portable pH meter (Mudder brand, United States of America, accuracy: ±0.3) in each subplot as an average of three measurements taken diagonally at equidistant points.

**Figure 2:**
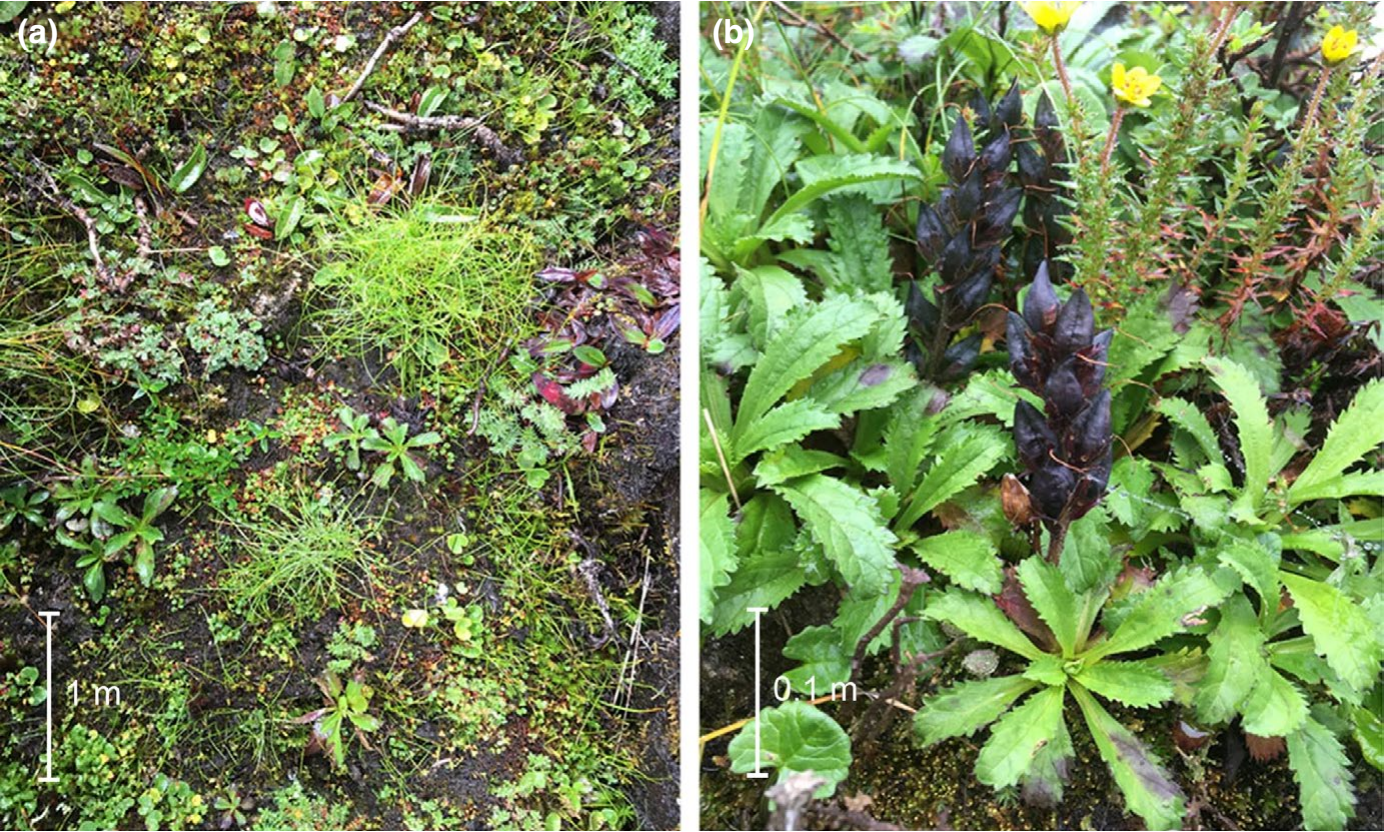
*Neopicrorhiza scrophulariiflora* growing in (a) heavily harvested and (b) unharvested habitats; an individual plant at fruiting stage is 5–25 cm tall. The approximate size of objects is indicated by the scale bars

Evidence of MAP harvesting and livestock grazing was confirmed by intensive observation in each subplot. In order to determine the magnitude of impact, subplots were further divided into four cells, each 0.25 m^2^ in size. We assigned a binary score to each cell based on whether there was evidence of disturbance (1) or not (0). At the subplot level, the scores given separately for each category of disturbance were combined for the four cells to obtain an overall score ranging from 0 (null) to 4 (very high). The following types of evidence were used in assessing the harvesting of *Neopicrorhiza*: the presence of left‐over rhizome fragments, uprooted plants, and holes or scars left after the excavation of plants (Figure 2). Assessment of grazing was based on the presence of pugmarks and trampling, bite marks, browsed plants, broken, defoliated or disorientated aerial parts and animal droppings.

### 2.6 Data analysis

Variation in ramet density, biomass, and environmental covariates among populations and between regions was tested by nonparametric Kruskal–Wallis one‐way ANOVA and Mann–Whitney *U* tests. A generalized linear mixed effects model (GLMM) was used to examine the effect of environmental factors on ramet density, and a linear mixed effects model (LMM) was used for analyzing rhizome biomass and tissue density. A negative binomial distribution was assumed for ramet density, and rhizome biomass and tissue density were transformed using the natural logarithm to make their residuals normally distributed. To control for intra‐class correlation caused by the nested design, “plot” was included as a random effect in models for ramet density. Similarly, a random effect of “subplot” was included in models for rhizome biomass and tissue density. Effects of region and population were treated as fixed nominal factors. All numeric environmental predictors (Table S2) were treated as covariates. These covariates were standardized (mean 0, standard deviation 1) to avoid erroneous conclusions when analyzing interactions between continuous variables and to facilitate the interpretation of variables and models (Bråthen & Lortie, 2016). We used qq‐plots to evaluate the properties of residuals in models based on different distribution assumptions (Zuur, Ieno, Walker, Saveliev, & Smith, 2009). In the case of ramet density, the primary additive model was prepared by starting with a complete model, including all fixed effects and the random plot effect. Then, the model was reduced successively by backward elimination, gradually removing predictors that were not significant (*p* ≥ 0.05). Based on the final model with only significant predictors, extended models including two‐way interactions between regions, harvest, and elevation were prepared. In the case of rhizome biomass and tissue density, the number of potential predictors was lower and a selection procedure was not applied. For each model, the Akaike information criterion (AIC) was calculated and compared with the null model. The model with the lowest AIC value was considered as the best model. To assess the validity of the selected model, we performed likelihood ratio tests comparing models with fixed effects to the null model. Pearson correlation analysis was used to examine co‐variation between response and predictor variables when more elaboration was required. The correlation analysis, Kruskal–Wallis and Mann–Whitney *U* tests were carried out in IBM SPSS 24 (IBM, 2016), and GLMM and LMM were prepared using the lme4 package in R version 3.5 (R Core & Team, 2017).

## 3 RESULTS

### 3.1 Variation in utilization pattern

We recorded both opportunistic (26%) and dedicated (74%) collectors in ANCA. About, 5% of MAP users interviewed (*n* = 165) did not harvest *Neopicrorhiza*, 90% harvested it both for trade and local healthcare, and the rest 5% (all traditional healers) collected it only for local health care. Among the MAP users, 7%, 57%, and 31% collected *Neopicrorhiza* before the growth season (May/June), after the growth season (August/September) and both before and after, respectively. A conservative estimate of the annual collection is that on average people from ANCA collected 0.5–3.5 kg (mean ± *SE* = 1.56 ± 0.06 kg) dry rhizomes per household for local health care and 35–465 kg (mean ± *SE* = 170.50 ± 6.64 kg) per household for trade.

We found no evidence of trade‐driven collection in the LNP region. In contrast to ANCA, all the MAP collectors interviewed in LNP were opportunistic, 87% (33 of the 38 interviewees) harvested *Neopicrorhiza*, and 88% of them told that the collection was for local health care. Only few users in LNP collected *Neopicrorhiza* during May/June (6%) and the majority collected in August/September (94%). Among the collectors, about 12% indicated willingness to sell the rhizomes should an opportunity arise, which is however unlikely due to the legal restrictions implied by the protected area system. Like in ANCA, people in LNP collected 1.0–3.0 kg (1.89 ± 0.09 kg) dried rhizome per household annually for local healthcare consumption.

### 3.2 Variation in plant density

**Figure 3:**
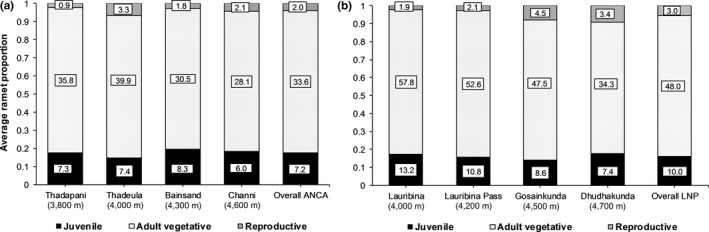
Population‐wise proportions of different life stages derived from ramet density (mean value (m^−2^) is included in each part of the columns) of *Neopicrorhiza scrophulariiflora* in two regions: (a) Api‐Nampa Conservation Area (ANCA) and (b) Langtang National Park (LNP), Nepal

Ramet density (m^−2^) varied within and between the regions (Figure 3). Importantly, for total ramet density (all stages combined), LNP populations were 1.5 times denser than ANCA populations (mean ± *SE* = 42.93 ± 3.35 for ANCA, 61.12 ± 3.83 for LNP, *p* < 0.0001). For individual stage classes, the densities in the two regions decreased in the same order from adult vegetative (33.60 ± 2.60 and 48.05 ± 3.07, *p* < 0.0001), over juvenile (7.28 ± 0.66 and 10.03 ± 0.77, *p* = 0.002) to reproductive (2.05 ± 0.31 and 3.03 ± 0.37, *p* = 0.006, in ANCA and LNP, respectively). In ANCA, only reproductive density varied significantly (*p* = 0.004) among populations and was highest in Thadeula and lowest in Thadapani (Figure 3 **)**. Similarly, in LNP juvenile density did not differ much among the populations, but adult vegetative density was highest in Lauribina and lowest in Dhudhakunda (*p* = 0.002), and reproductive density was highest in Gosainkunda and lowest in Lauribina (*p* = 0.019, Figure 3). The population structure in terms of ramet stage class composition clearly deviated between the study regions (*p* = 0.023, Kolmogorov–Smirnov test) with greater reproductive ramet proportion in LNP (0.053 ± 0.006) than in ANCA (0.033 ± 0.004, *p* = 0.011). By contrast, the proportions of ramets in other stage classes did not vary between the regions (Figure 3). Also, ramet composition did not vary substantially across populations within each region (*p* = 0.267 for ANCA and 0.656 for LNP).

### 3.3 Variation in rhizome biomass

**Figure 4:**
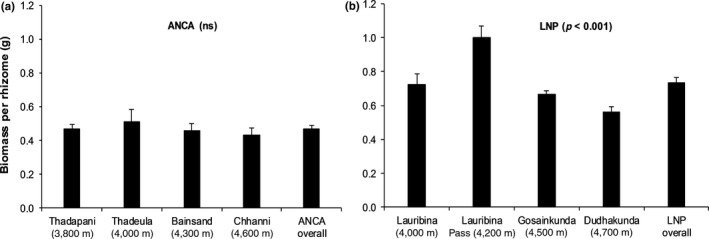
Population‐wise ramet biomass (Mean ± *SE*) of *Neopicrorhiza scrophulariiflora* in (a) Api‐Nampa Conservation Area (ANCA) and (b) Langtang National Park (LNP) in Nepal. The variation between populations was tested using the Kruskal–Wallis test (*p*‐values stated in brackets)

Ramet girth (cm) was higher in ANCA (mean ± *SE* = 0.211 ± 0.008) than in LNP (0.176 ± 0.006, *p* < 0.001). Likewise, mean rhizome volume (cm^3^) was also higher in ANCA (0.36 ± 0.05) than in LNP (0.26 ± 0.02, *p* = 0.023). On the other hand, the rhizome tissue density (g/cm^3^) was considerably higher in LNP (5.53 ± 0.37) than in ANCA (2.66 ± 0.22, *p* < 0.001). In addition, the mean rhizome biomass (dry weight in g) was about 1.5 times higher in LNP (0.74 ± 0.03) than in ANCA (0.47 ± 0.02, *p* < 0.001). In ANCA, mean rhizome biomass did not deviate between the populations **(**Figure 4a**)**. Moreover, within LNP, the biomass appeared to be highest for the mid‐elevation population, Lauribina Pass at 4,200 m and, otherwise, it decreased with increasing elevation (Figure 4b**)**. In ANCA, an average of 2,136 dried rhizomes (range: 1,964–2,310) were needed to obtain one kilogram; while in LNP, an average of 1,358 rhizomes (range: 1,001–1,780) were required per kg. In both regions, the number of individuals needed to obtain one kilogram of dried rhizome generally increased with elevation.

### 3.4 Effect of environmental factors on plant density and biomass

The environmental variables recorded in the sample plots varied within and between regions (Table S2). Major differences between regions were observed for ground cover variables and disturbances. Shrub, rock, and moss/lichen cover were higher in LNP than in ANCA. Cover of herbs and graminoids was higher in ANCA than in LNP. The average scores of harvesting and grazing were remarkably higher in plots studied in ANCA than in LNP (Table S2).

**Figure 5:**
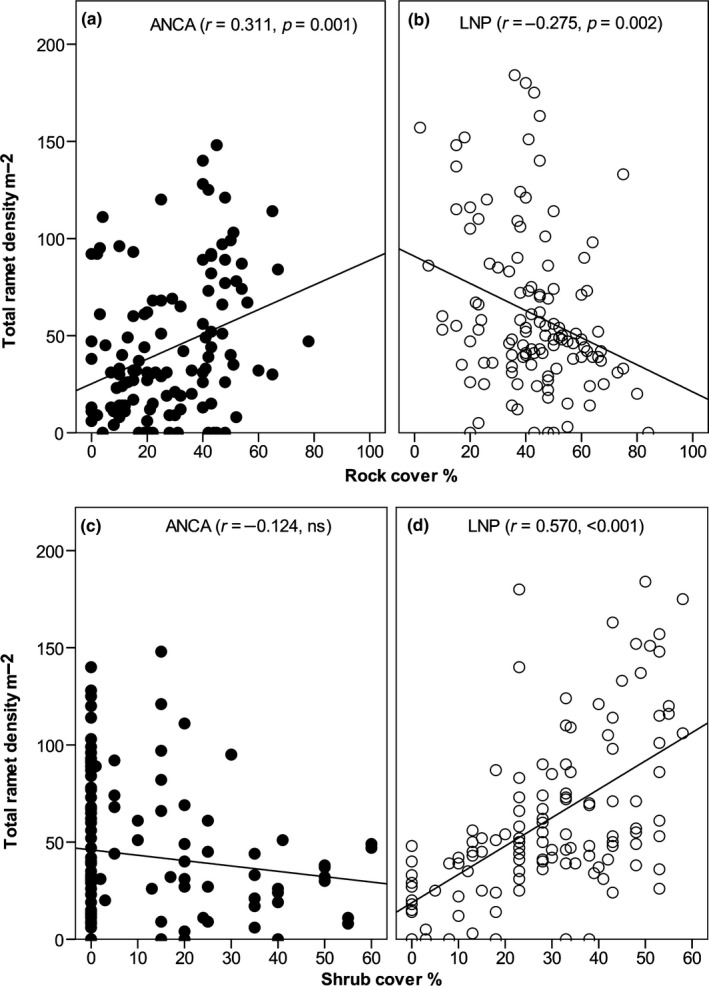
Relationship between total ramet density (all stages, m^−2^) and (a, b) rock cover, and (c, d) shrub cover for *Neopicrorhiza scrophulariiflora* in Api‐Nampa Conservation Area (ANCA) and Langtang National Park (LNP), Nepal

Out of twelve variables (including “population”) studied (Table S2), only nine were used in GLMM after removing confounded variables. Moss/lichen cover was confounded with shrub cover, and bare ground cover was confounded with herb and graminoid cover. Shrub height revealed several missing values and so, it was excluded in the model. Generalized linear mixed effects model identified five variables that exhibited a clear association with plant density (Table 1). Rock cover, in general, did not show any notable effect on plant density when data from both regions were treated together. However, when data were treated separately for each region, rock cover was found to be associated positively with plant density in ANCA (Pearson correlation, *r* = 0.323, 0.281, 0.320 and 0.311 for juvenile, adult vegetative, reproductive and total ramet density, respectively; *p* < 0.010; Figure 5a). By contrast, in LNP the corresponding relationship was negative (*r* = −0.303 and −0.275 for adult vegetative and total ramet density, respectively; *p* < 0.010; Figure 5b).

**Table 1:** Generalized linear mixed effect models expressing the effects of environmental factors on ramet density (m^−2^) of *Neopicrorhiza scrophulariiflora* in Api‐Nampa Conservation Area (ANCA) and Langtang National Park (LNP), Nepal

Explanatory variable	Juvenile		Adult vegetative		Reproductive		Overall	
	Parameter estimate	Standard error	Parameter estimate	Standard error	Parameter estimate	Standard error	Parameter estimate	Standard error
ANCA								
Intercept: Thadapani 3,800 m	2.117***	0.180	3.695***	0.153	0.295	0.272	3.923***	0.157
Thadeula 4,000 m	−0.232	0.254	0.22	0.215	0.874*	0.342	0.205	0.22
Bainsand 4,300 m	−0.162	0.254	−0.206	0.213	0.382	0.364	−0.172	0.218
Chhanni 4,600 m	−0.213	0.255	−0.243	0.222	0.203	0.361	−0.258	0.227
LNP								
Lauribina 4,000 m	0.264	0.253	0.16	0.221	−0.24	0.367	0.202	0.226
Lauribina Pass 4,200 m	−0.259	0.263	−0.212	0.230	−0.676	0.369	−0.195	0.236
Gosainkunda 4,500 m	−0.169	0.271	−0.213	0.224	0.117	0.344	−0.189	0.231
Dhudhakunda 4,700 m	−0.346	0.274	−0.467*	0.229	−0.574	0.359	−0.414	0.235
Cover substrate								
Shrub cover	0.230***	0.069	0.281***	0.069	0.294***	0.084	0.260***	0.071
Graminoid cover	−0.237***	0.064			−0.368***	0.098		
Edaphic								
Soil pH	0.157*	0.072						
Disturbance								
Harvest	−0.390***	0.072	−0.556***	0.072	−0.871***	0.088	−0.570***	0.074
Grazing	0.335***	0.07	0.402***	0.071	−0.143	0.08	0.384***	0.072
AIC: Full	1,483.931		2,210.60		865.266		2,331.222	
AIC: Null	1,530.20		2,269.50		973.90		2,385.51	
Observations (*n*)	240.00		240.00		240.00		240.00	

Note

Models were estimated assuming a negative binomial distribution and “plot” was included as a random factor. Levels of significance are indicated by the symbols: *p* < 0.0001 “***,” *p* < 0.001 “**,” and *p* < 0.05 “*.”

In the combined model, shrub cover also appeared to have a positive effect on ramet density (Table 1). When data were treated separately for each region, it emerged that increasing shrub cover was associated with increasing ramet density in LNP (Figure 5d) but not in ANCA. For individual stage classes in LNP, shrub cover showed a weaker positive relation with reproductive ramet density (*r* = 0.269, *p* = 0.003) than with juvenile (*r* = 0.398, *p* < 0.001) and adult vegetative (r = 0.579, *p* < 0.001) ramet density. By contrast, in ANCA, the shrub cover showed a negative relationship with ramet density (Figure 5c), but the relationship was only significant for reproductive ramet density (*r* = −0.229, *p* = 0.012).

Among all environmental variables studied, harvest disturbance had a negative effect on ramet density for all stage classes, whereas the effect of grazing was positive for juvenile and adult vegetative ramet densities (Table 1). A significant interaction between harvest impact and region further indicated that the negative effect of harvest on plant density was stronger in ANCA, and the effect of harvest was subtle for the LNP populations (Table 2). The weak positive effect of harvest on ramet density in LNP (positive interaction in Table 2) for all vegetative stages could be linked to the narrow range of harvest intensities.

**Table 2:** Generalized **l**inear mixed effect models expressing the effect of environmental factors on ramet density (m^−2^), ramet biomass (g), and tissue density (g/cm^3^) of *Neopicrorhiza scrophulariiflora* in Api‐Nampa Conservation Area (ANCA) and Langtang National Park (LNP), Nepal

Explanatory variable	Juvenile		Adult vegetative		Reproductive		Total ramets		Rhizome biomass		Rhizome tissue density	
	Parameter estimate	Standard error	Parameter estimate	Standard error	Parameter estimate	Standard error	Parameter estimate	Standard error	Parameter estimate	Standard error	Parameter estimate	Standard error
Region												
Intercept: ANCA	1.874***	0.126	3.441***	0.103	0.333*	0.161	3.684***	0.104	−0.889****	0.056	0.814***	0.093
LNP	0.410*	0.173	0.454**	0.140	−0.076	0.226	4.126***	0.099	0.497***	0.084	0.215	0.139
Harvest	−0.667***	0.094	−0.757***	0.100	−1.164***	0.129	−0.762***	0.101	−0.279***	0.053	−0.353***	0.087
Elevation	−0.264	0.135	−0.360**	0.111	−0.435**	0.168	−0.354**	0.112	−0.118	0.060	0.251*	0.101
Interaction												
LNP*Harvest	0.805***	0.123	0.820***	0.131	0.296	0.177	0.796***	0.133	0.079	0.086	−0.044	0.144
LNP*Elevation	0.134	0.178	0.204	0.146	0.524*	0.218	0.211	0.148	−0.078	0.081	0.144	0.136
AIC: Full	1,481.752		2,209.50		865.266		2,326.979		234.20		657.40	
AIC: Null	1530.20		2,269.50		973.90		2,385.51		295.80		717.50	
Observations (*n*)	240.00		240.00		240.00		240.00		294.00		294.00	

Note

Parameters were estimated assuming a negative binomial distribution for density, and natural log transformation was used to control for the skewed distribution of biomass and tissue density. A random effect of “Plot” was included for ramet density, and for biomass and tissue density of rhizome “Subplot” was included as a random factor in the models. Levels of significance are indicated by the symbols: *p* < 0.0001 “***,” *p* < 0.001 “**,” and *p* < 0.05 “*.”

The harvest impact showed different association patterns with rock and shrub cover in the two regions. In LNP, the harvest score exhibited no clear relation with shrub cover (*p > *0.050), whereas the relationship was positive in ANCA (*r* = 0.326, *p* < 0.001). Contrary to this, in ANCA, the harvest impact score was negatively related with rock cover (*r* = −0.268, *p* = 0.003); whereas, in LNP, the relation was not significant. The positive relationship between harvest impact score and shrub cover further indicated that shrubland habitats in ANCA were preferred collecting sites for *Neopicrorhiza* harvesters.

In general, while fitting variables in GLMM (Table 2), we detected a lower value of AIC when elevation (AIC = 2,282.85) was included in models instead of populations (2,287.90, against null model = 2,385.51). This may indicate that part of the variability among the populations can be ascribed to differences in elevation (Table 2 **)**. Elevation generally had negative parameter estimates, indicating that ramet density gradually decreases with increasing elevation. In contrast to the other stages, reproductive ramet density in LNP was weakly positively related to elevation (Table 2 **)**. Otherwise, the effect of elevation on ramet density was less negative in LNP than in ANCA, which could be related to the better protection characterizing LNP. Moreover, harvest was heavier at low elevations than further uphill (*r* = −0.252, *p* = 0.006, for ANCA and *r* = −0.367, *p* = 0.001 for LNP), presumably reducing ramet densities at low elevations.

**Figure 6:**
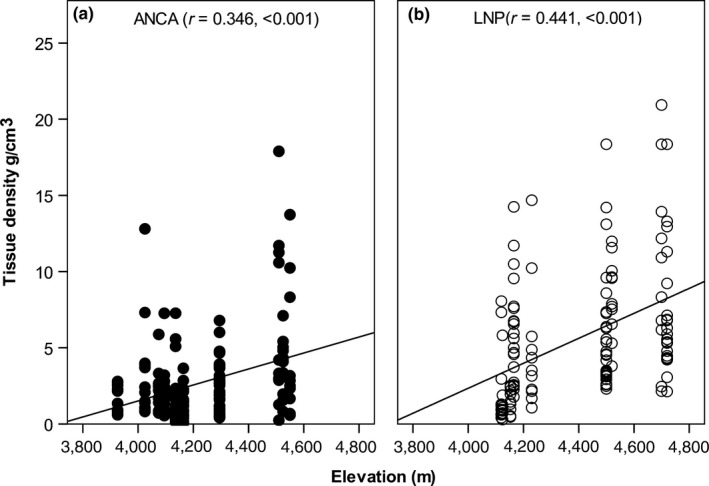
Relationship between elevation and rhizome tissue density of *Neopicrorhiza scrophulariiflora* in (a) Api‐Nampa Conservation Area (ANCA) and (b) Langtang National Park (LNP), Nepal

Rhizome girth decreased with increasing elevation but this effect was more prominent in LNP (*r* = −0.490, *p* < 0.001) than in ANCA (*r* = −0.162, *p* = 0.040). Rhizome volume was not correlated with elevation in ANCA, but in LNP, it decreased with increasing elevation (*r* = −0.591, *p* < 0.001). Harvest negatively affected rhizome biomass in both regions and this effect was slightly lower in LNP than in ANCA (Table 2). We did not observe an effect of elevation on biomass (Table 2), but rhizome tissue density increased with elevation in both regions (Table 2). This indicates that tissue density was more sensitive to environmental variation along the elevation gradient than biomass. Additionally, this may suggest that older ramets with denser tissues were more prevalent at higher elevation, especially in populations in LNP (Figure 6a,b).

## 4 DISCUSSION

### 4.1 Utilization practices and pressure on wild MAP resources

We observed less harvest pressure in LNP (which is protected) than ANCA (which is open‐access region) and encountered a lower number of *Neopicrorhiza* users in LNP where all harvest was for local use. By contrast, in ANCA a greater number of users harvested *Neopicrorhiza* for trade than for local use, and the amount harvested per household was higher than reported in earlier studies from the Himalayas (e.g., Ghimire et al., 2005; Uniyal, Uniyal, & Rawat, 2011). For example, Pyakurel et al. (2018) estimated the trade of *Neopicrorhiza* from ANCA to be 18.55 tonnes in fiscal year 2014/2015. The amount harvested for local healthcare was similar in the two regions but, due to the heavy harvest for trade in ANCA, increases in harvest in this region may seriously threaten its *Neopicrorhiza* populations.

In Nepal, *Neopicrorhiza* is one of the high‐valued MAPs of the alpine region with a century‐long trade history (Olsen, 2005; Olsen & Larsen, 2003). In recent years, the exploitation of herbal and fungal MAPs has become a significant challenge for sustainable resource management in the Himalayas (Cunningham et al., 2018; Negi, Pant, Joshi, & Bohra, 2014). Presently, due to lucrative markets and surging prices, the collection of the highly valued parasitic fungus *Ophiocordyceps* has exceeded the total income from the harvest of all other MAPs (Negi et al., 2014; Pouliot et al., 2018). However, when valuable MAPs become scarce, collectors start collecting other highly valued products (Lange, 2004; Mulliken, 2000).

Since livelihood options in ANCA are constrained by its rugged terrain and remoteness, people supplement their income primarily by harvesting MAPs and other forest products (Pouliot et al., 2018; Pyakurel et al., 2018). In contrast, LNP is an attractive tourist destination in Nepal, in part because it features an important pilgrimage site (the sacred Gosainkunda lake), which is visited by tens of thousands of devotees every year (Shakya, 2016). People in LNP therefore supplement their income by engaging in tourism and hotel business instead of harvesting and trading forest and environmental products.

The broad geographic distribution of *Neopicrorhiza* in the Himalayas implies that this species is an easily accessible economic resource to many rural people (Olsen, 2005; Olsen & Larsen, 2003). The cash income earned by selling high‐value medicinal plants, including *Neopicrorhiza* can be an appreciable supplement to local subsistence (Olsen, 2005). Conveniently, in ANCA, the traditional practice of MAP harvesting is legally permitted through the Management Council, formed based on Protected Area Management Regulation, which issues collection and transportation permits for all legal environmental products (HMG/N, 1978). All national parks in Nepal located at high elevation are managed under the Himalayan National Park Regulation (HMG/N, 1978) which also includes a provision to issue harvesting permits to selected MAPs within the park boundary. However, in LNP, permits to collect and trade MAPs have been provided only for *Ophiocordyceps* (personal communication with a conservation officer, 26 July 2018). It therefore stands to reason that, respondents in LNP might be less likely to report commercial collection of *Neopicrorhiza* than respondents in ANCA, where such collection is permitted. Nevertheless, some studies indicate that illegal harvest of MAPs could occur in both regions due to geographical remoteness and lack of regular invigilation mechanisms (Pyakurel et al., 2018; Shrestha, Prasai, Shrestha, Shrestha, & Zhang, 2014; Uprety et al., 2011).

### 4.2 Variation in plant density and biomass

Our observations show that the variations in ramet density and rhizome biomass of *Neopicrorhiza* correlate with harvest intensities and physical habitat variables. The effect of commercial harvesting appeared critical for plant density, rhizome size, and biomass in ANCA. Results indicated that the plant density and biomass are 1.5 times higher in LNP than in ANCA, likely demonstrating that the former is better protected than the latter. Compared to LNP, in ANCA 1.6 times more ramets must be collected to obtain one kilogram (dry weight) of rhizomes. Based on our annual observations, a conservative estimate is that commercial collectors in ANCA collect approx. 364,000 rhizomes (average: 2,136 rhizomes/kg) to achieve their harvest of 170 kg dry weight. This estimate suggests that commercial harvest may have a stronger negative effect on *Neopicrorhiza* populations than other medicinal rhizomatous Himalayan species like *P. kurrooa*, where only 200–500 rhizomes are needed per kg of dry mass (Rai, Prasad, & Sharma, 2000; Uniyal et al., 2011). Given the strong harvest pressure, we suggest an action plan to be prepared for ANCA to mitigate the risk of over‐collecting *Neopicrorhiza* rhizomes in this region. A large variation in ramet density between study regions was observed for adult stages with lower density in ANCA than in LNP. Furthermore, a greater proportion of reproductive ramets in LNP than in ANCA may be an additional indication of the heavy collection pressure that affects *Neopicrorhiza* populations in ANCA. Moreover, Ghimire et al. (2005), Ghimire et al. (2008) stated that alpine MAPs, including *Neopicrorhiza,* should be left unharvested for at least 5–10 years to allow sufficient time for individuals to grow and reproduce between the harvests.

Plant density and biomass decreased with increasing elevation, indicating how environmental stresses (e.g., lower temperature, shorter growing time) constrain growth at high elevations (He, Xue, Gao, Wang, & Wu, 2017). However, in LNP reproductive ramet density increased weakly with elevation (Table 2) but, otherwise, the overall effect of elevation was weakly negative in LNP and clearly negative in ANCA. The increase in reproductive ramet density in LNP along the elevation could reflect a reduced harvesting pressure with increasing elevation in combination with a tendency of plants to be more likely to grow to reproductive maturity when growing in less disturbed conditions. However, in ANCA, the negative effect of human disturbance on *Neopicrorhiza* was evident, also for the reproductive stage and regardless of elevation. This finding is consistent with Rusterholz, Kissling, and Baur (2009)*,* who observed that sexual reproduction in the rhizomatous clonal herb *Anemone nemorosa* increased with declining human disturbance.

### 4.3 Major habitat determinants

In LNP, the shrub cover and moss/lichen cover showed a positive relationship with *Neopicrorhiza* density (*r* = 0.787, *p* < 0.0001). Hence, it could be suggested that shrub vegetation with substantial moss/lichen ground cover maintains a high moisture level that favors *Neopicrorhiza* populations and leads to increased density. Positive effects of shrub facilitation, such as reduced evapotranspiration, improved thermoregulation, and soil amendment by litter accumulation and decomposition have been reported in previous studies (e.g., Ballantyne & Pickering, 2015). However, in LNP the shrub cover generally decreased with increasing elevation. Based on the density and biomass estimates in LNP, we suggest low elevation populations (<4,500 m) are likely more resilient to low‐intensity harvest, in part due to facilitative effects offered by shrub cover.

However, in ANCA, rock cover showed a positive relationship with plant density (Figure 5a), whereas the relationship between shrub cover and plant density was not significant. Our interviews with MAP collectors revealed that their impression was the opposite, as they believed that, compared to other habitats, shrubland habitats had higher biomass of *Neopicrorhiza* per unit area, which allowed them to collect larger amounts within a given period of time (data not shown). Commercial collectors usually harvest intensively in order to maximize yield while minimizing effort and time. Hence, they prefer to harvest in dense populations where high quantities of rhizomes are available (Ghimire, McKey, & Aumeeruddy‐Thomas, 2004). We therefore suggest that the observed higher density of *Neopicrorhiza* in rocky habitats in ANCA may be due to the preference of harvesters for the shrubland habitats at lower elevations.

In our study, grazing showed a positive effect on ramet density except for the reproductive stage (Table 1). Our field observations indicated that *Neopicrorhiza* is unpalatable to herbivores, which could be linked to secondary metabolites, such as terpenoids, cucurbitacin, kutkisterol, and steroids present in the plant (Li et al., 2014). Additionally, in alpine meadows, grazing tends to decrease competition for light without altering functional diversity of foliar traits (Niu et al., 2016). Hence, the removal of species other than *Neopicrorhiza* by grazing reduces competition for light and provides the species with suitable conditions for vegetative regeneration. Indeed, the guerrilla growth form of *Neopicrorhiza* allows it to multiply as soon as it finds open space (Ghimire et al., 2005; Humphrey & Pyke, 1998).

Clonal growth is a successful strategy for persistence of plant populations in stressful environment like those of the Himalayas (Dvorský et al., 2013, 2015; Herben, Šerá, & Klimešová, 2014). Based on the parameter estimates obtained for juvenile and adult vegetative ramet densities it appears that for given levels of harvest and elevation, densities were higher in LNP than ANCA (Table 2). Ghimire et al. (2005) also found higher values of *Neopicrorhiza* density at low levels of harvesting. The plagiotropic growth of the plant enhances its ability to adapt to extreme environmental conditions where the competition from orthotropic plants is less important (Ghimire et al., 2005; Katayama et al., 2015). The ramets are only partly autonomous, which enables them to benefit from nutrients (nitrogen, phosphorus, potash, and urea) and photosynthates (sugars, amino acids, and hormones) made available by already existing ramets of the same genet (Lei, 2010). Previous studies (Evette et al., 2009; Johansen, Wehn, & Hovstad, 2016; Lei, 2010) found that the budding potential and ramet production of clonal herbs in disturbed environments is higher than in undisturbed environments. Consequently, the clonal growth and fugitive establishment of *Neopicrorhiza* could partly remediate the impact of low‐intensity harvest in disturbed populations.

## 5 CONCLUSION

The present study provides baseline evidence on plant utilization systems, population differentiation, and habitat conditions of *Neopicrorhiza* across a conservation gradient. Both plant density and biomass indicate that a low harvest intensity had no adverse effect on the populations, although quantification of the optimum level of harvest remains to be done and is therefore an aim of ongoing research. Market‐driven harvesting and premature exploitation are the most important issues for long‐term management. The high number of harvesters in the open‐access region appeared to have a strong negative impact on *Neopicrorhiza* populations, whereas in the protected region such clear effects were not observed. Shrubland habitats exhibited high plant densities in the protected region (LNP), but in the open‐access region (ANCA), heavy exploitation seemed to occur in such habitats and so, a higher plant density occurred in rocky areas instead. To ensure that the number of reproductive individuals left in each harvested population is sufficient to maintain population viability, we propose regulating harvest such as by introducing rotational harvest systems and in situ management of *Neopicrorhiza* populations. Additionally, we suggest regulating management through implementing community monitoring of harvest, involving local users, collectors, and park officials. Finally, we recommend analyzing the effects of harvest on populations dynamics to estimate suitable limits of annual harvest and develop strategies for sustainable management.

## CONFLICT OF INTEREST

The authors declare that no competing interests exist.

## AUTHOR CONTRIBUTIONS

MRP and SKG designed the experiment; MRP collected data; MRP, HM, BBS, and SKG analyzed the data and wrote the manuscript.

## Data Availability

Data used for ecological analysis are presented in the paper and the supplementary material. Data that identify human participants in the study will not be made publicly available. However, such data can be made available upon request to the authors.
