# Supplementary material for: Harvest effects on density and biomass of Neopicrorhiza scrophulariiflora vary along environmental gradients in the Nepalese Himalayas
Source: Ecol Evol. 2019 Jun 19;9(13):7726–40. doi: 10.1002/ece3.5355 (PMC6635918; doi:10.1002/ece3.5355)
Supplement: Supplementary file 1 [file ECE3-9-7726-s001.docx]

**SUPPLEMENTARY MATERIAL**

**Title of paper: Harvest effects on density and biomass of *Neopicrorhiza scrophulariiflora* vary along environmental gradients in the Nepalese Himalayas**

Mukti Ram Poudeyal^1, 2^*^*^*, Henrik Meilby^2^, Bharat Babu Shrestha^1^ and Suresh Kumar Ghimire^1^*^*^*

^1^Central Department of Botany, Tribhuvan University, Kirtipur, Kathmandu, Nepal

^2^Department of Food and Resource Economics, University of Copenhagen, Rolighedsvej 23, 1958 Frederiksberg C, Denmark

^*^**Correspondence**

Mukti Ram Poudeyal

Central Department of Botany, Tribhuvan University, Kirtipur, Kathmandu, Nepal and Department of Food and Resource Economics, University of Copenhagen, Rolighedsvej 23, 1958 Frederiksberg C, Denmark

Email: muktipoudeyal@gmail.com

Phone number: 977-9841056732

Suresh Kumar Ghimire

Central Department of Botany, Tribhuvan University, Kirtipur, Kathmandu, Nepal

Email: sk.ghimire@cdbtu.edu.np

Phone number: +977- 1123 4567

**Table S1**

Populations of *Neopicrorhiza scrophulariiflora* sampled in Api-Nampa Conservation Area and Langtang National Park, Nepal with topographical features, geographical coordinates, climate and nature of habitat.

| **Population** | **Harvest level** | **Mean elevation (m asl.)** | **Mean slope in degrees** | **Aspect** | **Geographical coordinates** | **Climate type** | **Dominant habitat type** |
| --- | --- | --- | --- | --- | --- | --- | --- |
| **Api-Nampa Conservation Area, North-West Nepal** | | | | |  |  |  |
| 1. Thadapani | High | 3800 | 39.00 | NE-NW | 29.97 N, 80.93 E | Lower alpine | Grassland and shrubland meadow |
| 1. Thadeula | Low | 4000 | 36.00 | NE-SW | 29.97 N, 80.95E | Alpine | Shrubland meadow, Moraine |
| 1. Bainsand | High | 4300 | 45.00 | NE-NW | 29.95 N, 80.96 E | Alpine | Grassland and shrubland meadow, Moraine |
| 1. Chhanni | Low | 4600 | 46.00 | NE | 29.95 N, 80.93 E | Upper alpine | Grassland and rocky area |
| **Langtang National Park, North-Central Nepal** | | | |  |  |  |  |
| 1. Lauribina | High | 4000 | 55.83 | NE-NW | 28.09 N, 85.37 E | Alpine | Grassland and Shrubland meadow |
| 1. Lauribina Pass | Low | 4200 | 47.83 | NW | 28.09 N, 85.39 E | Alpine | Shrubland meadow, Moraine |
| 1. Gosainkunda | High | 4500 | 56.67 | NE | 28.07 N, 85.42 E | Upper alpine | Grassland and Shrubland meadow, Moraine |
| 1. Dudhakunda | Low | 4700 | 46.17 | NE | 28.09 N, 85.42 E | Upper alpine | Shrubland meadow and rocky boulders |

**Table S2**

Mean (± SD) values of environmental factors among populations of *Neopicrorhiza scrophulariiflora* in Api-Nampa Conservation Area and Langtang National Park, Nepal. Variation of medians among populations in each region was tested using Kruskal Wallis tests; levels of significance: p<0.001‘***’, *p*<0.01‘**’ and *p*<0.05‘*’. Study regions were compared based on Mann-Whitney *U* tests (N = 240).

| **Environmental variables** | **Api-Nampa Conservation Area (ANCA)** | | | | |  | **Langtang National Park (LNP)** | | | | | |  |
| --- | --- | --- | --- | --- | --- | --- | --- | --- | --- | --- | --- | --- | --- |
|  | Thadapani (3800 m) | Thadeula (4000 m) | Bainsand (4300 m) | Channi (4600 m) | ANCA overall |  | Lauribina (4000 m) | Lauribina Pass (4200 m) | Gosainkunda (4500 m) | | Dhudhakunda (4700 m) | LNP overall | Region difference (*p* value) |
| **Ground cover estimate** |  |  |  |  |  |  |  |  |  | |  |  |  |
| Herb cover % | 39.03±3.37 | 35.57±2.95 | 46.10±4.07 | 31.40±2.05 | 38.03±1.65* |  | 29.1±2.02 | 22.63±1.47 | 35.30±2.83 | | 12.87±1.49 | 24.98±1.26*** | <0.001 |
| Graminoid cover % | 15.03±2.11 | 8.40±1.08 | 9.93±1.92 | 15.50±1.16 | 12.22±0.85*** |  | 11.63±1.14 | 9.43±0.74 | 9.50±0.97 | | 7.53±1.50 | 9.53±0.57*** | <0.050 |
| Rock cover % | 21.10±3.07 | 34.53±3.45 | 22.20±2.76 | 33.83±3.14 | 27.92±1.64** |  | 39.03±2.26 | 41.13±2.16 | 35.53±3.31 | | 54.23±3.14 | 42.48±1.51*** | <0.001 |
| Bare ground cover % | 5.90±1.10 | 9.23±1.76 | 10.00±1.57 | 11.73±1.27 | 9.22±0.74* |  | 3.13±0.79 | 3.20±0.65 | 5.47±1.39 | | 6.33±1.59 | 4.53±0.59 | <0.001 |
| Moss/Lichen cover % | 18.93±2.64 | 12.27±1.52 | 11.77±3.07 | 7.50±1.03 | 12.62±1.16*** |  | 17.07±1.32 | 23.60±1.34 | 14.20±1.6 | | 19.03±2.02 | 18.48±0.85*** | <0.001 |
| Shrub cover % | 20.33±3.70 | 10.87±2.58 | 10.17±3.18 | 2.50±1.28 | 10.97±1.51** |  | 28.73±2.9 | 38.43±1.82 | 25.23±3.17 | | 23.70±3.24 | 29.03±1.50** | <0.001 |
| Shrub height m | 0.71±0.07 | 0.61±0.05 | 0.51±0.10 | 0.46±0.14 | 0.61±0.04 |  | 0.57±0.02 | 0.52±0.02 | 0.43±0.02 | | 0.37±0.02 | 0.48±0.01*** | <0.010 |
| **Solar radiation** |  |  |  |  |  |  |  |  |  | |  |  |  |
| Potential annual direct incident radiation (PADIR) | −0.84±0.11 | −0.77±0.10 | −0.97±0.10 | −0.96±0.02 | −0.88±0.04** |  | −1.17±0.05 | −0.97±0.03 | | −1.19±0.05 | −0.94±0.06 | −1.07±0.03*** | <0.001 |
| **Edaphic factor** |  |  |  |  |  |  |  |  |  | |  |  |  |
| Soil pH | 6.47±0.05 | 6.63±0.03 | 6.49±0.04 | 6.58±0.04 | 6.54±0.02* |  | 6.25±0.06 | 6.38±0.04 | 6.14±0.05 | | 6.23±0.05 | 6.25±0.03** | <0.001 |
| **Disturbance score** |  |  |  |  |  |  |  |  |  | |  |  |  |
| Grazing effect (0…4) | 2.93±0.19 | 2.47±0.16 | 2.77±0.14 | 2.17±0.16 | 2.58±0.08* |  | 2.77±0.23 | 2.70±0.19 | 2.03±0.24 | | 1.33±0.22 | 2.21±0.12** | 0.049 |
| Harvest effect (0…4) | 3.23±0.22 | 2.80 ± 0.25 | 3.13±0.20 | 2.17±0.21 | 2.83±0.12** |  | 2.56±0.24 | 1.73±0.23 | 1.56±0.20 | | 1.03 ± 0.19 | 1.73±0.12*** | <0.001 |
